# The Role of Payoff Valence on Voting: Egalitarian for Gains and Selfish for Losses

**DOI:** 10.3389/fpsyg.2021.737225

**Published:** 2021-11-24

**Authors:** Carlos Alós-Ferrer, Michele Garagnani, Jaume García-Segarra

**Affiliations:** ^1^Zurich Center for Neuroeconomics, Department of Economics, University of Zurich, Zurich, Switzerland; ^2^LEE & Department of Economics, University Jaume I, Castellón de la Plana, Spain

**Keywords:** gains, losses, framing, payoff valence, plurality voting, approval voting

## Abstract

We study how payoff valence affects voting behavior on the distribution of monetary outcomes framed as gains or losses in a group when using standard *plurality* voting (PV) procedures and when using *approval* voting (AV). The latter method allows the subjects to approve of as many alternatives as they wish and has been shown to eliminate the incentives to vote strategically. For both methods, we observe that voters express higher support for egalitarian allocations (and lower support for selfish options) when sharing gains than when sharing losses. Moreover, the average number of approved alternatives per ballot is higher when distributions are framed in terms of gains than when they are framed in terms of losses. We also discuss under which circumstances the shift in voting behavior is more likely to produce changes in the electoral outcome. The results suggest that framing manipulations (payoff valence) can significantly impact voting behavior.

## 1. Introduction

An extensive theoretical and empirical literature has discussed the virtues and vices of different voting methods (e.g., Riker, 1982). However, little is known about how actual voting behavior under different methods reacts to different frames. Taking two prominent voting methods as a benchmark, i.e., plurality voting (PV) and approval voting (AV), we investigate how voting behavior is affected by one of the most prominent framing manipulations, namely payoff valence (gains vs. losses). The motivation is that alternatives in elections, from investment plans in firms to political programs, can often be framed in different forms depending on the reference point, for instance describing possible consequences as gains or as losses with respect to a hypothetical ideal.

Commonly-used voting methods in actual political elections are often based on PV. Under this method, voters are asked to report only their most-preferred alternative, i.e., the maximum of their respective preferences, hence disregarding all other information contained in those preferences. In contrast, under AV, each voter is allowed to vote for (or “approve of”) as many alternatives as wished (Brams and Fishburn, 1978). This voting method requires that voters reveal which alternatives are acceptable, i.e., each voter needs only to report the alternatives she approves of. The alternative with the highest number of approvals then becomes the winner of the election. The properties of this method have been studied both theoretically and empirically. A key advantage is that the method eliminates incentives to vote strategically (Brams and Fishburn, 1978; Alós-Ferrer and Buckenmaier, 2019). Intuitively, under PV, voters might be tempted by “wasted vote” arguments to not to vote for their most-preferred alternative if it is believed to have low chances of winning, while under AV, there is no reason not to approve of it (possibly together with other alternatives). Several empirical studies have tested the performance of AV in the field by conducting large-scale field experiments during actual elections (Laslier and Van der Straeten, 2008; Alós-Ferrer and Granić, 2010, 2012; Baujard and Igersheim, 2010) and in the lab (Laslier, 2010; Bassi, 2015; Granić, 2017). This method has been adopted (in sometimes slightly altered forms) by many scientific, engineering, and professional societies, including, among others, the American Mathematical Society, the U.S. National Academy of Sciences, the election of Secretary-General of the United Nations, and the Social Choice and Welfare Society. It has also been proposed as an alternative for political elections and is currently used for municipal elections in Fargo (North Dakota) and Saint Louis (Missouri).

### 1.1. Related Literature

The literature on individual decision making has shown that framing the same choice in terms of gains or losses has strong effects on behavior. According to Prospect Theory (Kahneman and Tversky, 1979), the typical behavioral pattern is that subjects are risk averse in the domain of gains but risk seeking in the domain of losses. Further, empirical evidence has shown that decision makers exhibit *loss aversion*, i.e., they are more sensitive to losses compared to gains (Kahneman and Tversky, 1984; Tversky and Kahneman, 1991) both for choices involving risk (Tversky and Kahneman, 1981; Novemsky and Kahneman, 2005) and uncertainty (Tversky and Kahneman, 1986). However, these studies involve individual decision making which does not affect others. Studies on decision making for others suggest that decisions made on behalf of others differ from those made for oneself (refer to e.g., Tunney and Ziegler, 2015; Füllbrunn et al., 2020), particularly interms of risk (Polman and Wu, 2020). For example, Zhang et al. (2017) found that risk-taking to avoid potential losses is reduced when deciding on behalf of others. Losecaat Vermeer et al. (2020) found increased risk-taking after incurring a loss as compared to a gain, but this effect was substantially reduced when choices were made on behalf of others. This suggests that loss aversion might be weaker when the involved losses are not those of the decision maker.

Few studies have examined the effect of framing in terms of gains vs. losses in interpersonal situations where outcomes affect both the individual and others. An exception is De Dreu and McCusker (1997), which shows that, when considering whether to contribute to a public good, people with a more prosocial disposition cooperate more in a loss frame, while people with a more individualistic orientation cooperate more in a gain frame. In the case of voting, most empirical studies examine only distributional allocations framed in terms of gains, and only few consider losses. In the context of coalition formation, Van Beest et al. (2005) found that loss aversion has heterogeneous effects depending on individual attitudes toward others. Individualistic decision makers are mostly motivated to minimize their losses since losses loom larger than gains. In contrast, other-regarding decision makers find it less appropriate to inflict losses on others. The latter effect is in line with the “do-no-harm” principle (Baron, 1995; Royzman and Baron, 2002). The idea is that subjects are more reluctant to cause losses to others (through action) than to reduce their gains. Field experiments analyzing framing effects on Online Voting Advice Applications (VAAs) have shown that wording questions positively vs. negatively in the VAAs systematically affects voter responses (Lee et al., 2016), in particular for voters with a low level of political sophistication (Kamoen et al., 2019). Recently, Lockwood and Rockey (2020) discussed how loss aversion from a reference point (status quo) influences bipartisan elections for the U.S. House of Representatives (challenger vs. incumbent) in terms of how candidates adjust policies.

### 1.2. The Present Research

In our experiment, participants faced equivalent elections, in groups of six voters per election, using both AV and PV methods, with the particularity that in half of the elections, outcomes were framed as gains and in the other half, they were framed as losses from a reference point. We induced preferences over alternatives using monetary payoffs and considered two different payoff profiles. Every payoff profile included individualistic, self-centered alternatives, and confronted them with socially desirable outcomes. Specifically, every election included an egalitarian alternative (yielding the same outcome for every voter) and a socially-efficient one (in the sense of maximizing the sum of payoffs). The latter was included since other-regarding preferences often include an efficiency concern (Charness and Rabin, 2002). In each election, both the egalitarian and the socially-efficient alternative avoided inflicting severe losses to other voters.

In accordance with previous literature (e.g., Van Beest et al., 2005), two opposite predictions arise. On the one hand, the other-regarding approach to loss aversion postulates that voters avoid causing losses to other subjects through their actions. Therefore, in line with the “do-no-harm” principle, they become reluctant to optimize their payoffs at the cost of actively harming others when outcomes are framed in terms of losses, a motivation that is absent (or at least diminished) if they are framed in terms of gains. Hence, there should be less support for selfish options in the loss domain than in the gain domain. On the other hand, the self-interested approach to loss aversion suggests that voters will mainly focus on their payoffs when voting in a loss frame and will try to minimize their losses before any other consideration. Since, under loss aversion, losses loom larger than gains, non-selfish options become less acceptable in a loss frame than in a gain frame, and hence, the opposite prediction arises, namely higher support for selfish options in the loss domain than in the gain domain. In other words, in this case, avoiding losses becomes more prominent, while the losses inflicted on others play no role. Our study addresses the empirical question of which of these hypotheses prevails in a voting context.

We also conjectured that payoff valence would have a significant incidence in the acceptance threshold in AV, and in particular, in the number of options approved of. Specifically, we expect more approvals under a framing on gains than under losses no matter which loss aversion approach finally prevails. This is clear under the “other-regarding approach,” because voters should be reluctant to administer losses through action and, thus, will generally refrain from approving alternatives harming other voters. However, this hypothesis could also be argued to be compatible with the “self-interested approach,” in the sense that if voters focus mainly on their payoff, they will be more wary of suboptimal alternatives under a loss frame than under a gain frame.

Our results show that payoff valence significantly influences the proportion of choices over alternatives. Under both PV and AV, the egalitarian alternative finds higher support under gains than under losses, and the opposite is true for self-interested options. Note that these are independent statements for AV (but not for PV) since under this method, approving of one option does not preclude approval of a different option. Therefore, the evidence supports the self-interested approach to loss aversion over the “do-no-harm” principle in our voting experiment. Regarding the acceptance threshold we observe that, as expected, voters approve of significantly more alternatives under a gain frame compared to a loss frame.

The study is organized as follows. Section 2 describes the experimental design. Section 3 presents the analysis of individual voting behavior for both payoff profiles under both voting methods and shows how the framing manipulation affected voting decisions and electoral outcomes. Section 4 concludes.

## 2. Experimental Design

Our sample comprises 141 participants (67 women) in a 2 within (voting method: AV vs. PV) × 2 between (treatment condition: gains vs. losses frame) design. We ran the experiment at the “Laboratori d'Economia Experimental” (LEE) in the University Jaume I of Castelló de la Plana (Spain) using z-Tree (Fischbacher, 2007). Participants were recruited from the student population of the university using the LEE proprietary recruitment system RECS. Three other participants were excluded from the analysis because they did not understand the instructions of the experiment or did not obey the rules of the lab^1^.

### 2.1. Procedures

The experimental procedures follow the basic design of voting experiments due to Forsythe et al. (1993, 1996) (refer to also Granić, 2017). We used monetary incentives in the form of payoff tables displaying the rewards from different election outcomes to induce preferences over alternatives (refer to right-hand side of Tables 1, 2). All payoffs were presented in terms of Experimental Currency Units (ECU) and converted at the end of the experiment at a fixed rate of EUR 0.20 per ECU. There were four available alternatives in each election, and all ties between two or more alternatives were broken randomly. The ballots were displayed on the screen, and voters could decide their choice anonymously. Empty ballots (abstention) were not allowed^2^. All participants were informed about the full payoff table in each election. Thus, they knew the induced preferences of all voters in the election. Under AV, voters could choose as many alternatives as wished in each ballot. The election winner was the alternative collecting the highest number of approvals. In contrast, under PV, voters had to choose one alternative only, and the winner was determined by the number of votes received.

**Table 1:** Society 1 in terms of gains and losses. Payoffs for gains and losses are numerically equivalent.

**Voter**	**Nr**.	**Induced preferences**	**Gains**					**Losses**				
**Type**			**A**	**B**	**C**	**D**	**Total**	**A**	**B**	**C**	**D**	**Total**
I	2	*A*≻*C*≻*D*≻*B*	80	30	60	55	225	−20	−70	−40	−45	−175
II	2	*B*≻*C*≻*D*≻*A*	20	90	60	55	225	−80	−10	−40	−45	−175
III	2	*D*≻*C*≻*A*≻*B*	55	30	60	80	225	−45	−70	−40	−20	−175
Total			155	150	180	190		−145	−150	−120	−110	

*The losses in every cell reflect detraction from an initial endowment of 100 Experimental Currency Units (ECU). Thus, the vertical totals (of three rows) are [gains = 300 minus losses] and horizontal totals (of four columns) are [gains = 400 minus losses]*.

**Table 2:** Society 2 in terms of gains and losses.

**Voter**	**Nr**.	**Induced preferences**	**Gains**					**Losses**				
**Type**			**A**	**B**	**C**	**D**	**Total**	**A**	**B**	**C**	**D**	**Total**
Type			A	B	C	D	Total	A	B	C	D	Total
I	2	*A*≻*D*≻*C*≻*B*	80	30	55	60	225	−20	−70	−45	−40	−175
II	2	*B*≻*C*≻*D*≻*A*	30	90	55	50	225	−70	−10	−45	−50	−175
III	2	*D*≻*A*≻*C*≻*B*	60	30	55	80	225	−40	−70	−45	−20	−175
Total			170	150	165	190		−130	−150	−135	−110	

*Payoffs for gains and losses are numerically equivalent. The losses in every cell reflect detraction from an initial endowment of 100 ECU. Thus, the vertical totals (of three rows) are [gains = 300 minus losses] and horizontal totals (of four columns) are [gains = 400 minus losses]*.

Participants were randomly allocated to different groups of six voters each. The group was fixed for the entire experiment. In each of the three sessions (each containing eight groups), half of the groups faced the elections framed in terms of gains, with payoff tables containing the number of participants of the ECUs would earn if each alternative won the election (see Tables 1, 2). The other half had the elections framed in terms of losses, with payoff tables displaying the number of ECUs to be subtracted from an endowment of 100 ECU to compute the final payoff of each alternative if it was the winner of the election (refer to Tables 1, 2). The resulting outcomes were numerically equivalent across treatments by design, and the only difference across treatments was the valence of payoffs.

In each treatment, two different payoff tables or *Societies* (inducing two different preference profiles) were presented. The difference concerns mainly the socially efficient outcome. In Society 1 (refer to Table 1), this outcome maximizes the sum of payoffs but creates high inequality, with only a minority benefiting from it. In Society 2 (refer to Table 2), the efficient outcome benefits a majority. In each round, each participant was assigned to one of three types. Those corresponded to three different preferences induced through the payoffs in the tables. That is, each table displayed three different voter types, each of which corresponded to exactly two voters in each voting group. The assignment to types was such that every voter made decisions for every type in every payoff table (in different voting rounds). Further, after six election rounds, participants faced a new voting method. Hence, each subject had to face elections using both voting methods. The order of methods was counterbalanced, with half of the groups (in each treatment) starting with AV and switching to PV after six voting rounds, and conversely for the other half. Therefore, subjects voted 12 times, one for each possible type and each preference profile under each voting method, with a new decision situation presented each round. Additionally, after the 12th election round, a preference elicitation mechanism was implemented, which has no impact on the results reported in this study (refer to Supplementary Materials).

To avoid learning, feedback, and repeated-game effects, election outcomes were not announced until the end of the experiment. That is, at the end of each election, participants went directly to the next round without knowing the previous round outcome^3^. At the end of the experiment, for each group, one of the 12 rounds was randomly selected, implemented, and paid. The average payoff was EUR 11.64, ranging from EUR 4 to EUR 18. Experimental sessions lasted around 55 min.

### 2.2. Power

To ensure a sufficient number of independent observations, we conducted a power analysis before the data collection. The minimum required power for detecting a medium effect size (*d* = 0.5) on the voting behavior with a Mann-Whitney-Wilcoxon (MWW) test was set to 0.80, yielding a sample size of *N* = 67 per condition. We obtained data from 72 individuals per condition for a total *N* = 141 after three exclusions (as shown above).

## 3. Results

We present the results separately for Societies 1 and 2. In each case, we first describe the preference profile in the society and then present the results for individual behavior using both voting methods. We then present a brief description of the results for the number of approvals. Each subsection below concludes with the results for actual electoral outcomes. We then report the analysis comparing behavior in the two Societies. Finally, as a robustness check, we report on regression analysis of individual voting behavior.

For the analysis of individual voting behavior below, an observation, or *voter support*, is the average behavior of an individual voter in all situations that apply (which, for PV, is the same as the proportion of times that the voter chooses that alternative). Recall that every individual participated in 12 different elections, six for each method, and three for each Society (one for each of the three types). Hence, for PV, the support of a voter for a given alternative (and in one of the societies) is the average of the three corresponding decisions, or, equivalently, the fraction of the time that the voter voted for that alternative (for instance, if a voter voted selfishly two times out of three, the support for the selfish option is 2/3). For AV, to facilitate the exposition and the comparison between voting methods, the voter support is the average of the normalized approval scores in all decisions that apply. The normalized approval score for an alternative is one divided by the total number of approvals cast by the voter in that ballot, provided the alternative was itself approved of, and zero otherwise. Some of the comparisons below, however, concern the decisions made when acting as a particular type or types, which correspondingly reduces the set of decisions averaged to obtain individual voter support (for instance, every individual made two decisions as EW under AV in each Society).

### 3.1. Society 1

This society corresponds to the preference profiles given in Table 1 (terms of gains and terms of losses from 100 ECU). Alternatives *A* and *B* are the selfish options that maximize individual payoffs for voters of type I and II, respectively. We speak of *Self Interest* whenever type I voted for *A* or type II voted for *B*. Alternative *C* corresponds to the egalitarian outcome. Alternative *D* is the socially-efficient outcome in the sense that the sum of payoffs is maximized (this alternative also maximizes the individual payoffs of voters of type III). The preference profile suggests a division of the electorate in to two clearly-differentiated groups. The socially-efficient alternative *D* creates inequality and divides the electorate into *Efficiency Losers* (*EL*), namely voters of types I and II, and *Efficiency Winners* (*EW*), namely voters of type III. Hence, in this society efficiency favors only a minority.

#### 3.1.1. Voting Behavior (Gains vs. Losses)

When analyzing voting behavior, we further focus on how the support for the selfish option is affected by the voting method and the framing of the alternatives in terms of gains vs. losses.

**Figure 1:**
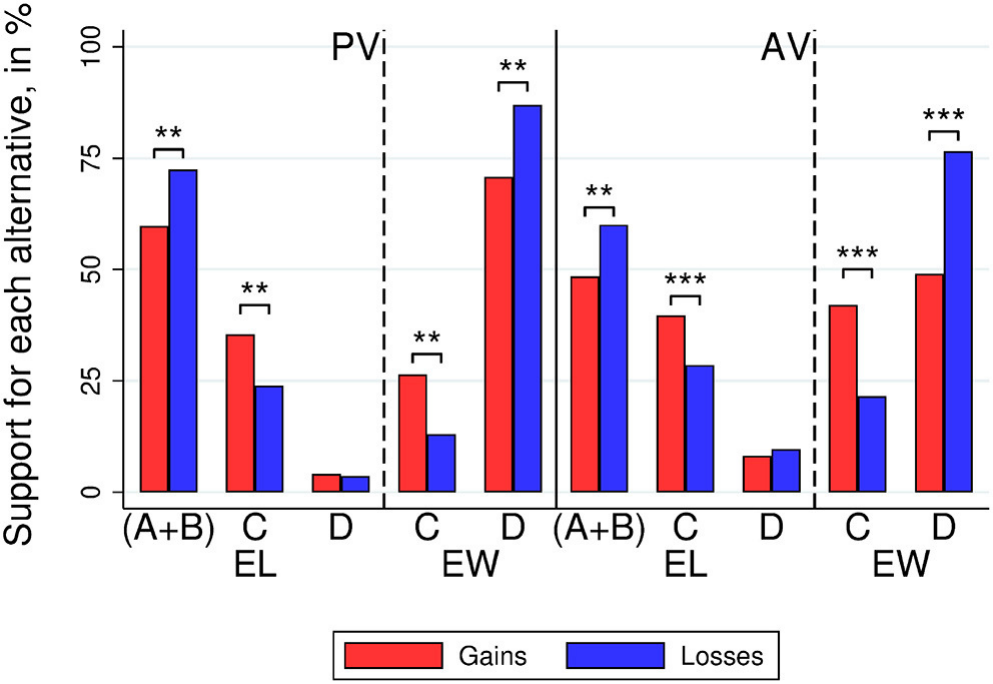
Society 1. Voter support for the different alternatives by treatment (gains vs. losses), voting method [plurality voting (PV) and approval voting (AV)], and voter group (Efficiency Winners and Efficiency Losers). A and B are the self-interested alternatives players of types I and II, respectively. Alternative C assigns equal payoffs to all voters, and D is a socially-efficient alternative. Stars refer to the significance of non-parametric tests (^**^p < 0.01^***^p < 0.001).

Figure 1 displays the average voter support over alternatives in Society 1, comparing results when outcomes were framed in terms of gains and losses, respectively, and splitting them by voting groups (*EL* and *EW*). Selfish alternatives (in the three elections of Society 1) were supported less when framed as gains than when framed as losses in both voting methods (MWW tests, *N* = 141; PV: 63.26 vs. 77.29%, *p* = 0.015; AV: 48.57 vs. 65.54%, *p* < 0.001). This is also true when considering only EL or EW separately. That is, EL (in the two elections where they had this role) supported selfish alternatives less when framed as gains than when framed as losses in both voting methods (MWW tests, *N* = 141; PV, *EL*: 59.72 vs. 72.46%, *p* = 0.035; AV, *EL*: 48.38 vs. 60.00%, *p* = 0.021). EW, in the only election round where they voted in this role, supported the socially-efficient alternative *D* (which maximizes their individual payoffs and, hence, is the selfish alternative for EW) less often under gains than under losses (MWW tests, *N* = 141; PV, *EW*: 70.83 vs. 86.96%, *p* = 0.031; AV, *EW*: 48.96 vs. 76.57%, *p* < 0.001).

Voters also supported the egalitarian alternative *C* more often under a gain frame than under a loss frame. MWW tests show that this finding holds for each group of voters (EW and EL) and each voting method (*N* = 141; PV, *EL*: 35.42 vs. 23.91%, *p* = 0.035; PV, *EW*: 26.39 vs. 13.04%, *p* = 0.044; AV, *EL*: 39.70 vs. 28.50%, *p* = 0.009; AV, *EW*: 42.01 vs. 21.50%, *p* < 0.001)^4^.

We can also investigate how framing effects interact with voter roles (EW vs. EL) and voting methods (PV and AV). First, each subject voted both in the roles of EW and EL. Subjects supported the selfish alternative more in the EW than in the EL role in both voting methods (MWW tests, *N* = 141; PV: 78.72 vs. 65.96%, *p* < 0.001; AV: 62.47 vs. 54.08%, *p* = 0.003). We computed the subject-level difference in support for the selfish alternative (EW minus EL) and found it to be larger under a loss frame than under a gain frame for AV (MWW test, *N* = 141; loss frame 16.55%, gain frame 0.58%, *p* = 0.008), but it is not statistically significant under PV (MWW tests, *N* = 141; loss frame 14.49%, gain frame 11.11%, *p* = 0.527).

Second, each subject voted in the same conditions using both PV and AV. Subjects supported the selfish option more under PV than AV (MWW test, *N* = 141; 70.21 vs. 56.88%, *p* < 0.001). Again, we computed the subject-level difference in support for the selfish alternative (PV minus AV) and found no significant differences between frames (MWW test, *N* = 141; gain frame 14.85%, loss frame 11.76%, *p* = 0.467).

#### 3.1.2. Number of Approvals

**Figure 2:**
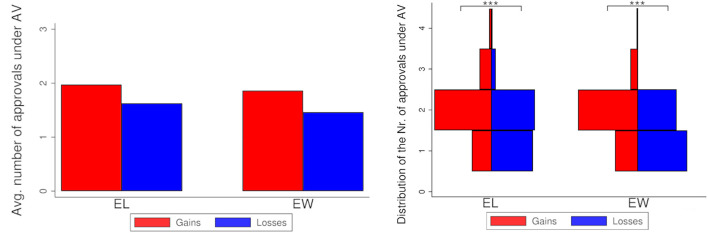
Society 1. **Left**: Number of approvals by treatment and group of voters. **Right**: Distribution of the number of approvals by treatment and group of voters. Stars refer to the significance of non-parametric tests (^***^p < 0.001).

Figure 2 (left-hand side) displays the average number of approvals per ballot under AV for both treatments, split by voters groups, and the distribution of the average number of approvals per ballot (right-hand side). Voters cast more approvals per ballot under gains than under losses, both for *EL* (average 1.97 vs. 1.62; MWW test, *N* = 141, *p* < 0.001) and for *EW* (average 1.86 vs. 1.46; MWW test, *N* = 141, *p* < 0.001).

#### 3.1.3. Electoral Outcomes

We now investigate whether the shift in individual behavior also translates into changes in electoral outcomes. Of course, electoral outcomes also depend on the specific societies we implement in the lab, and in particular on the fact that types were equally represented.

**Figure 3:**
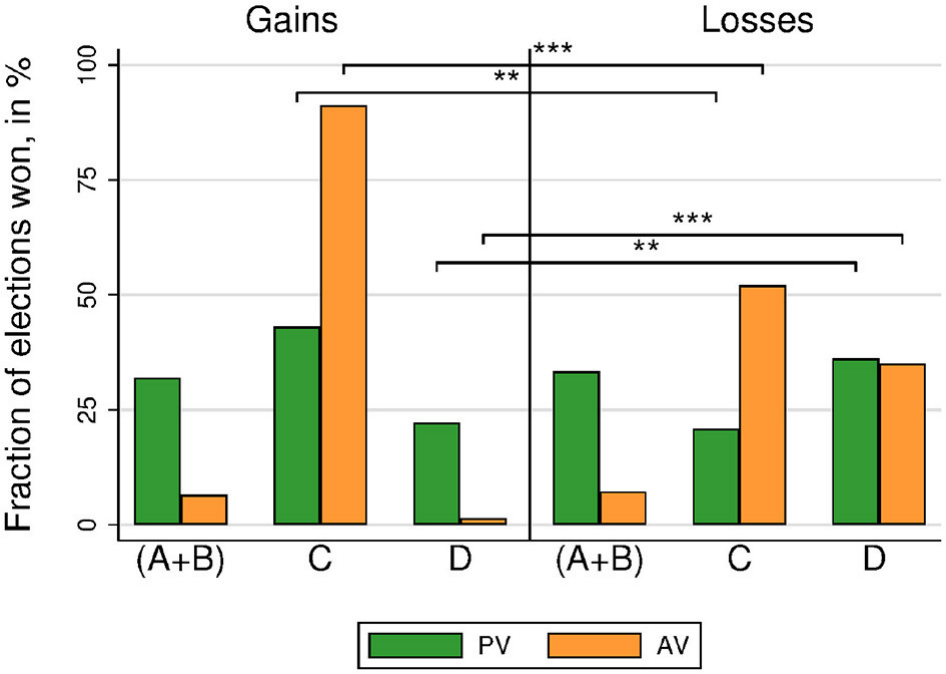
Society 1. Election winners (including tied winners) by voting method and treatment. Stars refer to the significance of non-parametric tests (^**^p < 0.01^***^p < 0.001).

Our experiment consisted of three sessions of four groups of six players voting six rounds for each method. This amounts to 144 elections (72 elections under each voting method)^5^. Figure 3 displays the percentage of elections won by each alternative. The egalitarian alternative *C* won more often when framed as gains than when framed as losses. Wilcoxon Signed-Rank tests (WSR) show that this finding holds both for both voting methods (*N* = 72; AV: 91.20 vs. 52.08%, *p* < 0.001; PV: 43.06 vs. 20.83%, *p* = 0.012). The socially-efficient alternative *D* (which maximizes payoffs for EW, i.e., type III voters) won more elections in a loss frame than in a gain frame (WSR, *N* = 72; AV: 1.39 vs. 34.95%, *p* < 0.001; PV: 22.22 vs. 36.11%, *p* = 0.043^6^. However, there is no difference in the percentage of elections won by the selfish alternatives *A* and *B* under gains and under losses (WSR, *N* = 72; AV: 6.48 vs. 7.18%, *p* = 0.343; PV: 31.94 vs. 33.33%, *p* = 0.964).

Focusing on the number of elections won allows for a straightforward comparison across voting methods. The egalitarian alternative *C* wins more often under AV than under PV, independently of the frame (WSR, *N* = 72; gains, 91.20 vs. 43.06%, *p* < 0.001; losses, 52.08 vs. 20.83%, *p* = 0.002). The socially-efficient alternative *D* wins less often under AV than under PV when the election is framed in terms of gains (WSR, *N* = 72; 1.39 vs. 22.22%, *p* < 0.001), but there is no difference in the case of losses (WSR, *N* = 72; 34.95 vs. 36.11%, *p* = 0.323).

### 3.2. Society 2

Society 2 corresponds to the preference profiles given in Table 2. As in the case of Society 1, alternatives *A* and *B* are the selfish options for type I and II voters, respectively, alternative *C* yields the egalitarian outcome, and alternative *D* is socially efficient in terms of the sum of payoffs and yields the maximum payoff for voters of type III. The difference is that, while in Society 1, the latter option only benefits a minority, in Society 2 it benefits a majority. Specifically, in Society 2, both type I and type III voters are EW, while only type II voters are EL (type I voters are better off if *D* is elected than if, say, the egalitarian outcome is implemented).

#### 3.2.1. Voting Behavior (Gains vs. Losses)

**Figure 4:**
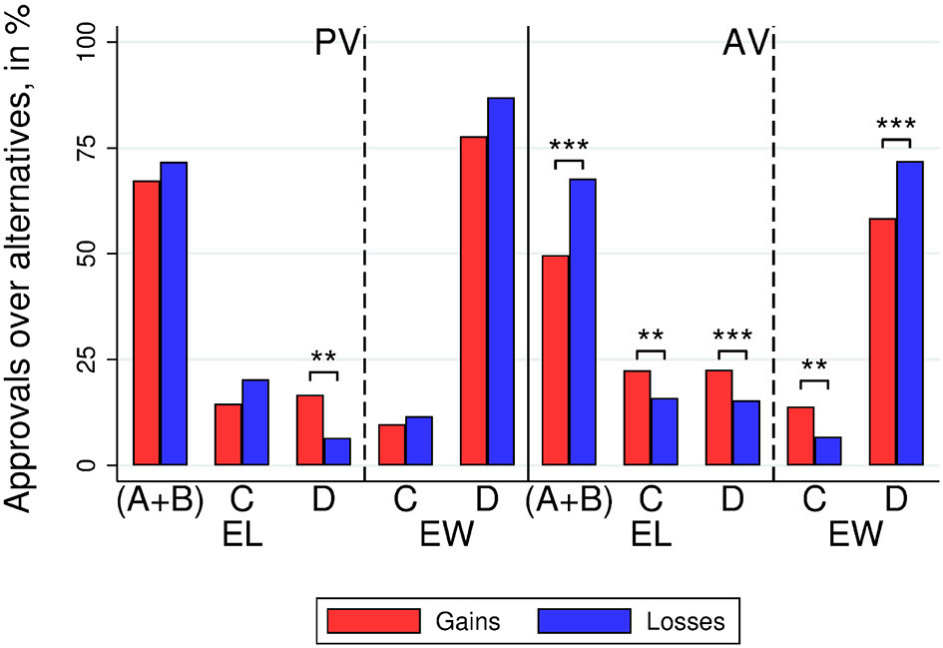
Society 2. Left: voter support for the different alternatives by treatment (gains vs. losses), voting method (PV and AV), and voter group (EW and EL). A and B are the self-interested alternative players of types I and II, respectively. Alternative C assigns equal payoffs to all voters, and D is a socially-efficient alternative. Stars refer to the significance of non-parametric tests (^**^p < 0.01^***^p < 0.001).

Figure 4 displays the voter support for the alternatives in Society 2 for each treatment (gains vs. losses), voting method (AV and PV), and voting groups (*EL* vs. *EW*). For AV, all results are as in Society 1. That is, in the three elections, this Society selfish alternatives were less supported when framed as gains than when framed as losses for AV (MWW test, *N* = 141; 52.55 vs. 69.16%, *p* < 0.001). This remains true when considering only EL or EW separately. That is, support for selfish options was lower for gains than for losses for EL (MWW, *N* = 141; *EL*: 49.65 vs. 67.75%, *p* < 0.001), and, analogously, support for the socially-efficient alternative *D* was also lower for gains than for losses for EW, for whom *D* maximizes payoffs (MWW, *N* = 141; *EW*: 58.33 vs. 71.98%, *p* = 0.007). For PV, however, there were no significant differences between frames (gains vs. losses) in the support for selfish options when considering all three decisions together (MWW test, *N* = 141; 70.83 vs. 76.81%, *p* = 0.182) and when considering EL or EW separately (MWW tests, *N* = 141, *EL*: 67.36 vs. 71.74%, *p* = 0.422; *EW*: 77.78 vs. 86.96%, *p* = 0.227).

Under AV, voters supported the egalitarian alternative *C* more often under gains than under losses for both *EL* and *EW* (MWW, *N* = 141, *EL*: 13.89 vs. 6.76%, *p* = 0.011; *EW*: 22.45 vs. 15.94%, *p* = 0.021). Under PV, the support for the egalitarian alternative *C* was not different between gains and losses for either group of voters (MWW, *N* = 141; *EL*: 14.58 vs. 20.29%, *p* = 0.402); *EW*: 9.72 vs. 11.59%, *p* = 0.930).

As in Society 1, in Society 2, subjects supported the selfish alternative more in the EW than in the EL role in both voting methods (MWW tests, *N* = 141; PV: 82.27 vs. 69.50%, *p* = 0.001; AV: 65.01 vs. 58.51%, *p* = 0.013). In contrast with Society 1, the difference in the support for the selfish alternative (EW minus EL) is not significantly different between frames (MWW tests, *N* = 141; AV: 8.68 vs. 4.23%, *p* = 0.248; PV: 10.42 vs. 15.22%, *p* = 0.749). Also as in Society 1, in Society 2, the support for the selfish option was larger under PV than under AV (MWW tests, *N* = 141; 73.76 vs. 60.68%, *p* < 0.001). The difference in the support for the selfish alternative (PV minus AV) was larger in the gain frame than in the loss frame (MWW test, *N* = 141; gain frame 18.29%, loss frame 7.65%, *p* = 0.047).

#### 3.2.2. Number of Approvals

**Figure 5:**
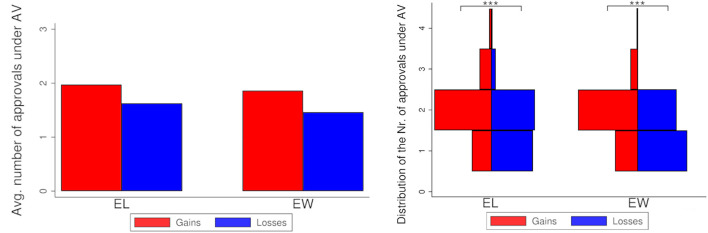
Society 2. **Left**: Number of approvals by treatment and group of voters. **Right**: Distribution of the number of approvals by treatment and group of voters. Stars refer to the significance of non-parametric tests (^**^p < 0.01^***^p < 0.001).

Figure 5 (left-hand side) displays the average number of approvals per ballot under AV for both treatments, split by voters groups, and the distribution of the average number of approvals per ballot (right-hand side). As in the case of Society 1, voters cast more approvals per ballot under gains than under losses, both for *EL* (average 1.86 vs. 1.55; MWW, *N* = 141, *p* < 0.001) and for *EW* (average 2.03 vs. 1.53; MWW, *N* = 141, *p* = 0.010).

#### 3.2.3. Electoral Outcomes

**Figure 6:**
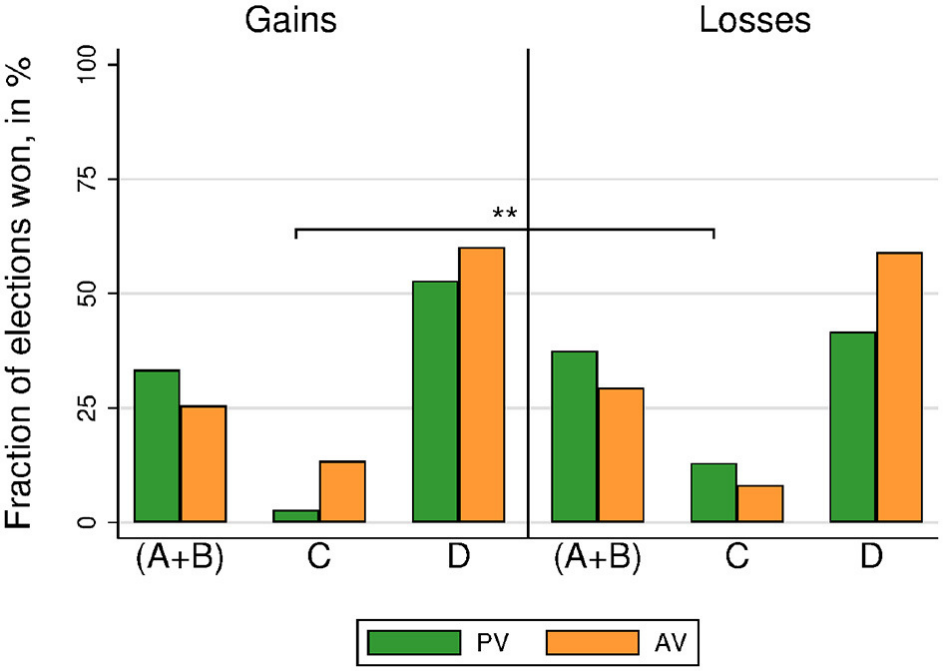
Society 2. Election winners (including tied winners) by voting method and treatment. Stars refer to the significance of non-parametric tests (^**^p < 0.01^***^p < 0.001).

Figure 6 displays the percentage of elections won by each alternative in Society 2. The percentage of elections won by every alternative is very similar across treatments (gains vs. losses), and also across methods (PV vs. AV) for each treatment. The only significant difference (all other *p*>0.1) is the comparison for the egalitarian alternative *C* under PV, which won less elections under gains than under losses (WSR, *N* = 72; 2.78 vs. 13.43%, *p* = 0.040)^7^. That is, in Society 2, shifts in voting behavior due to framing are not of sufficient magnitude to materialize in significant shifts in electoral outcomes.

### 3.3. Voting Behavior and Framing Effects Between Societies

We now investigate how individual behavior differs across the two Societies. The same subjects voted 12 times in total, six in each Society, with three rounds for each voting method and Society. However, the framing manipulation was between subjects. Selfish alternatives were less supported in Society 1 than in Society 2 (MWW test, *N* = 141; 63.54 vs. 67.22%, *p* = 0.003). This effect is present for both voting methods (MWW tests, *N* = 141; PV: 70.21 vs. 73.76%, *p* = 0.051; AV: 56.88 vs. 60.68%, *p* = 0.015). To investigate whether this effect is also influenced by the framing of the alternatives, we computed the subject-level difference in the support for selfish alternatives between societies (Society 2 minus Society 1). There are no significant differences across frames for either voting method (MWW tests, *N* = 141; PV: gain frame 7.41%, loss frame −0.48%, *p* = 0.138; AV: gain frame 3.97%, loss frame 3.62%, *p* = 0.818).

### 3.4. Regression Analysis

Our data forms a perfectly balanced panel with 12 decisions per participant, with six for each voting method. Hence, we rely on random-effects panel regressions on the voting (or approval) decision. This allows us to control for a variety of variables that might affect voting behavior and their interactions. We focus on the support of the selfish alternative, as the analyses concerning the other options would be correlated.

Table 3 displays the results of random-effects probit regression on the decision to vote for the selfish alternative under PV. That is, the dependent variable takes the value one if the subject voted for the selfish alternative, and zero otherwise. The results of the regression analysis confirm the non-parametric analyses. Model 1 investigates the main effect of the framing treatment. The loss frame dummy is positive albeit marginally significant, indicating a trend for higher overall support for the selfish alternative when alternatives are framed as losses than gains. In Model 2, we introduce a dummy for the preference profile (Society 2) and its interaction with the treatment. The loss frame dummy remains positive and significant, indicating higher support for the selfish alternative under losses than gains in Society 1. However, there is no significant treatment effect in Society 2, as indicated by the linear combination test (Loss Frame × Society 2 + Loss Frame), which is not significant. The dummy Society 2 is positive and significant, indicating higher support for the selfish alternative under a gain frame in Society 2 compared to Society 1. In Model 3, we further introduce a dummy for the voter group (EL) and its interaction with the treatment. EL votes for the selfish alternative less than EW independently of the frame as shown by the negative and significant Eff.Losers dummy for gains and by the negative and significant coefficient of the linear combination test (Loss Frame × Eff.Losers + Eff Losers) for losses.

**Table 3:** Random effect probit regressions on the probability of voting for the selfish alternative in plurality voting (PV).

**Selfish alternative**	**Model 1**	**Model 2**	**Model 3**
Loss frame	0.516^*^	0.683^**^	1.013^***^
	(0.264)	(0.290)	(0.369)
Society 2		0.320^**^	0.325^**^
		(0.155)	(0.158)
Loss frame × society 2		-0.341	-0.348
		(0.233)	(0.240)
Eff. losers			-0.493^***^
			(0.172)
Loss frame × eff. losers			-0.406
			(0.272)
Constant	0.754^***^	0.602^***^	0.965^***^
	(0.184)	(0.198)	(0.243)
Loss frame × society 2 + loss frame		0.342	0.665
		(0.291)	(0.369)
Loss frame × eff. losers + eff. losers			-0.899^***^
			(0.213)
*N*	846	846	846
*BIC*	838.308	847.491	832.702
χ^2^	3.820^*^	8.052^**^	32.380^***^

*Standard errors in parentheses*,

* *p < 0.1*,

** *p < 0.05*,

*** *p < 0.01*.

Table 4 displays the results of random-effect ordered-probit regression on the decision to approve of the selfish alternative in AV. For consistency with the previous analyses, the dependent variable is the normalized approval score, which is not binary and, hence, requires an ordered-probit regression. Model 1 investigates the main effect of the framing treatment. The loss frame dummy is positive and significant, indicating higher overall support for the selfish alternative when alternatives are framed as losses than when they are framed as gains under AV. In Model 2, we introduce a dummy for the preference profile (Society 2) and its interaction with the treatment. The loss frame dummy remains positive and significant, indicating higher support for the selfish alternative under losses than under gains in Society 1. The linear combination test (Loss Frame × Society 2 + Loss Frame) is also positive and significant, indicating that, also in Society 2, there is a significant treatment effect in the same direction of the other Society. The dummy Society 2 is not significant, indicating that the support for the selfish alternative is not significantly different (under a gain frame) in Society 2 compared to Society 1. In Model 3, we further introduce a dummy for the voter group (EL) and its interaction with the treatment. EL approve of the selfish alternative less than EW independently of the frame as shown by the negative and significant Eff.Losers dummy for gains, and the negative and significant coefficient of the linear combination test (Loss Frame × Eff.Losers + Eff.Losers) for losses.

**Table 4:** Random-effects ordered probit regressions on the probability of approving of the selfish alternative in approval voting (AV).

**Selfish alternative**	**Model 1**	**Model 2**	**Model 3**
Loss frame	0.919^***^	0.902^***^	1.211^***^
	(0.217)	(0.233)	(0.273)
Society 2		0.137	0.140
		(0.111)	(0.112)
Loss frame × society 2		0.040	0.040
		(0.168)	(0.169)
Eff. losers			-0.267^**^
			(0.120)
Loss frame × Eff. losers			-0.407^**^
			(0.183)
Constant	1.343^***^	1.354^***^	1.461^***^
	(0.234)	(0.236)	(0.254)
Loss frame × society 2 + loss frame		0.945^***^	1.251^***^
		(0.234)	(0.274)
Loss frame × eff. losers + eff. losers			-0.674^***^
			(0.140)
*N*	846	846	846
*BIC*	1847.120	1857.028	1841.700
χ^2^	17.909^***^	21.269^***^	47.220^***^

*Standard errors in parentheses*,

** *p < 0.05*,

*** *p < 0.01*.

## 4. Discussion

We find that manipulating the payoff valence in terms of gains or losses from a reference point significantly affects voting behavior, in the sense of increasing the support of the selfish alternative under losses (and generally increasing the support of equality under gains). These findings support the prediction suggested by the self-interested approach to loss aversion. That is when sharing losses in this voting context, subjects are mainly concerned about minimizing their losses and, consequently, they support the selfish alternative less often (and the egalitarian alternative more often) under a gain frame than under a loss frame. In other words, our results align with Losecaat Vermeer et al. (2020), but are contrary to the interpretation of Van Beest et al. (2005), and suggest that loss aversion might play a limited role when considering the losses of others.

Beyond this observation, the framing effect on voting behavior depends on the voting method and on whether the efficient outcome coincides with the egalitarian alternative or not. In particular, people tend to be more selfish under PV than under AV independently of the framing of the alternatives and the payoff structure. At the same time, they tend to be more supportive of the selfish alternative when the efficient outcome benefits a majority (Society 2) than when these alternatives are distinct. The latter effect is independent of the voting method and the framing. We further observe that, on the one hand, when the efficient outcome benefits the majority, voting behavior is more influenced by the framing (independently of the voting method used) for those subjects in the role of EW than for EL. On the other hand, when efficiency and equity differ, there is a stronger effect of the framing of alternatives on PV than on AV, but there are no significant differences depending on whether voters are EW or EL.

Regarding the number of approvals, voters cast more approvals per ballot under gains than under losses in both Societies. This supports our conjecture that payoff valence should influence the acceptance threshold in AV. This might be because voters are reluctant to administer losses through an explicit action and, thus, generally refrain from approving alternatives harming other voters.

The results regarding electoral outcomes are mixed. The shifts in individual voting behavior that we observe for both voting methods in Society 1 are sufficient to induce corresponding shifts in actual electoral outcomes in this society. However, this is not true for Society 2, even though we do observe shifts in individual behavior at least for the case of AV, in particular regarding the support for selfish alternatives. Although this is speculative at this point, this suggests that the degree of inequality created by socially-efficient outcomes might interact with behavioral shifts due to the framing of alternatives.

In conclusion, our study contributes to the understanding of the psychological determinants of voting behavior and suggests that the framing of alternatives, and especially payoff valence, can affect both individual voting behavior and electoral outcomes.

## Data Availability Statement

All materials (data, experiment's program, and data analysis code) are available on OSF at https://osf.io/n5kbc/.

## Ethics Statement

Ethical review and approval was not required for the study on human participants in accordance with the local legislation and institutional requirements. The patients/participants provided their written informed consent to participate in this study.

## Author Contributions

CA-F planned the project. MG and JG-S collected the data. CA-F, MG, and JG-S designed the experiment, analyzed data, and wrote the manuscript. All authors contributed equally to the project.

## Funding

Research was financed by Grant AL1169-2 of the German Science Foundation (Deutsche Forschungsgemeinschaft, DFG).

## Conflict of Interest

The authors declare that the research was conducted in the absence of any commercial or financial relationships that could be construed as a potential conflict of interest.

## Publisher's Note

All claims expressed in this article are solely those of the authors and do not necessarily represent those of their affiliated organizations, or those of the publisher, the editors and the reviewers. Any product that may be evaluated in this article, or claim that may be made by its manufacturer, is not guaranteed or endorsed by the publisher.

## Footnotes

^1^One student repeatedly used the phone to text during the experiment and asked if it was possible to leave the room at any point. Two others were not able to correctly answer the control questions even after the experimenter directly explained the crucial information to them. All three participants completed the experiment. The results do not change if their decisions are included in the analysis.

^2^Under AV one could interpret approving of all alternatives as an abstention. In our entire dataset, this happened only 15 times (1.77% of all 141×6 AV observations).

^3^In a laboratory voting game, Esponda and Vespa (2014) show that, when feedback is provided the number of non-strategic votes falls significantly. Thus, to elicit the voters' support for each alternative (i.e., voting behavior) as cleanly as possible and isolate the effect of the payoff valence on the one-shot voting decision, we deliberately refrained from providing feedback after each election.

^4^Note that tests for the support of different alternatives are not independent, and are included for completeness. For PV, voting for one alternative precludes voting for other alternatives, hence vote totals add up to a constant. A similar point applies to AV because approvals are normalized. However, note that these tests involve different (albeit correlated) variables, and hence multiple testing corrections are not necessary.

^5^For the comparison of electoral outcomes, we consider the entire dataset, that is, we do not exclude the groups of the three subjects who were excluded from the individual-behavior analysis.

^6^The number of winners is computed by adding 1/2 in case of a tie between two alternatives, etc. Similar conclusions are obtained if tied winners are removed from the analysis, except for the test for the socially-efficient alternative in the case of PV, which is not significant.

^7^This comparison misses significance if we remove tied winners from the analysis.
