# Supplementary material for: The Role of Payoff Valence on Voting: Egalitarian for Gains and Selfish for Losses
Source: Front Psychol. 2021 Nov 24;12:737225. doi: 10.3389/fpsyg.2021.737225 (PMC8653795; doi:10.3389/fpsyg.2021.737225)
Supplement: Supplementary file 1 [file Data_Sheet_1.pdf]

Supplementary Materials - The Role of Payoff Valence on Voting

Carlos Alós-Ferrer [1,*], **Michele Garagnani** [1] **and Jaume García-Segarra** [2]

[1] *Zurich Center for Neuroeconomics (ZNE), Department of Economics, University of Zurich. Blümlisalpstrasse 10, 8006 Zurich, Switzerland.*
[2] *LEE & Department of Economics, University Jaume I. Av. Vicent Sos Baynat, s/n 12071 Castelló de la Plana, Spain.*

Correspondence*:
Corresponding Author
carlos.alos-ferrer@econ.uzh.ch

1 SUPPLEMENTARY ANALYSIS

1.1 Strategic Voting: Induced Sincerity

An important question in the (empirical) voting literature is to what extent do voters engage in strategic considerations when voting, e.g. by misrepresenting their own preferences supporting a less-preferred alternative which has better chances of winning (Laslier, 2010). Strategic voting occurs when voters cast insincere ballots. Under PV, a ballot is sincere if the voter votes for the most-preferred alternative. Under AV, a ballot is sincere if the voter prefers each of the approved alternatives to each of the disapproved ones (Brams and Fishburn, 1978). This means that there are no gaps between what they voted for and their preferences (De Sinopoli et al., 2006; Laslier, 2009), i.e., if a voter casts a vote under AV containing alternatives A and D, sincerity means that the two most-preferred options must indeed be A and D. If the preferences were, say, $A \succ C \succ D \succ B$, a ballot containing only alternatives A and D would not be sincere, since the voter approves of D but not of C even though $C \succ D$.

We now examine sincerity under the assumption that the preferences are the ones induced by our payoff tables, i.e. the rankings given by the own, individual monetary payoffs. Hence, we speak of *induced sincerity*. In the next subsection, as a robustness check, we repeat the analysis under alternative, *elicited* preferences.

Specifically, we now examine the question of whether induced sincerity is affected by the framing in terms of gains or losses. Figure 1 (left-hand side) displays the percentage of sincere ballots according to the induced preferences, distinguishing treatments (gains vs. losses) and voting methods. In Society 1, and under PV, sincerity is higher under losses (48.31%) compared to gains (39.64%; MWW, $N = 141$, $p = 0.015$). However, there is no significant difference under AV (losses, 54.35%; gains, 52.08%; MWW, $N = 141$, $p = 0.517$). For Society 2, under PV sincerity is not significantly different between losses (48.01%) and gains (44.27%; MWW, $N = 141$, $p = 0.182$), but under AV sincerity is higher for losses (43.48%) than for gains (37.04%), although the difference is only marginally significant (MWW; $N = 141$, $p = 0.077$). Hence, we find (weak) evidence that a framing in the loss domain might sometimes increase sincerity.

1.2 Strategic Voting: Elicited Sincerity

In voting experiments, preferences are typically induced through monetary payoffs (Davis and Holt, 1993; Kagel and Roth, 2016). However, some authors have questioned this procedure. On the one hand,

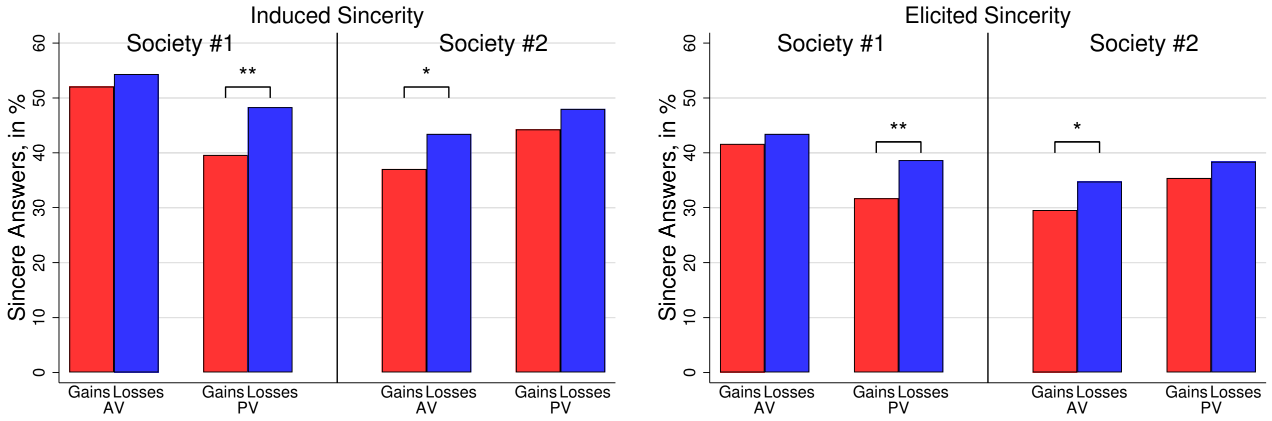

Figure 1. Induced and elicited sincerity by preference profile, treatment, and voting method.

an extensive literature on social preferences (Fehr and Schmidt, 1999; Bolton and Ockenfels, 2000; Charness and Rabin, 2002) suggests that people often exhibit many other-regarding preferences which are not maximized by the highest individual monetary payoff. Thus, the preferences induced by a payoff table might differ from the monetary ranking in the table. On the other hand, voters might have non-consequentialist motivations. Shayo and Harel (2012) argue that, when the probability of being pivotal is small enough, *expressive voting* can dominate in voting behavior, i.e., "voters may care about the act of voting itself, even if it does not affect the electoral outcome" (Shayo and Harel, 2012). Thus, if the probability of being pivotal is small, voting might for instance reflect increasing support for egalitarian alternatives simply because voters assume that their decision is inconsequential for the actual outcome. Even though the probability of being pivotal is relatively high in our experiment (compared to, e.g., political elections), a similar argument could be made because only one of the elections is randomly selected for the actual remuneration of subjects. Thus, in principle, non-consequentialist motivations could also play a role in our setting.

For these reasons, and following Alós-Ferrer and Buckenmaier (2021), our experiment included a method to elicit the voters' true preferences. This was done by including an additional task after finishing the 12 rounds of elections analyzed in the main text. The elicitation method uses a random dictator mechanism designed to elicit preferences over alternatives for each payoff profile (see Alós-Ferrer and Buckenmaier, 2021). This mechanism was a further election where a single voter would be randomly selected and this voter's decision would be implemented, independently of the decisions of others. That is, subjects were asked to choose the alternative that they would want to implement if they were selected to have their will enforced (hence a "random dictator mechanism"). To elicit preferences over all alternatives and not just the most-preferred one, subjects were informed that for each alternative, there was a small probability (5%) that this alternative could not be implemented. Hence, in a second step subjects were asked to decide a second alternative to implement in case their first choice was not feasible, and so on. Thus subjects provided a complete ranking over alternatives.

We now repeat the analysis in the previous section relying on these elicited preferences. Hence, we analyze sincerity with respect to the elicited preferences, or *elicited sincerity* (Figure 1, right-hand side). The results, however, are identical to the ones obtained under induced sincerity. In Society 1, under PV, sincerity is higher under losses (38.65%) compared to gains (31.71%; MWW, $N = 141$, $p = 0.016$), but

there is no difference under AV (losses 43.48%, gains 41.67%; MWW, $N = 141$, $p = 0.517$). In Society 2, under PV sincerity is not significantly different between loses (38.41%) and gains (35.42%; MWW, $N = 141$, $p = 0.182$), but under AV sincerity is higher for losses (34.78%) than under gains (29.63%; MWW, $N = 141$, $p = 0.078$).

Finally, we compare induced sincerity and elicited sincerity by computing the number proportion of elections (out of six) for each individual and voting method that the actual decision was sincere according to either preference. Elicited sincerity is systematically higher than induced sincerity for each combination of treatments, voting methods, and societies (all $p < 0.0001$). However, as can be seen in Figure 1, the differences are relatively small. For PV, average induced sincerity is 44.99%, compared to an elicited sincerity of 35.99%. For AV, average induced sincerity is 46.69%, compared to an elicited sincerity of 37.35%.

1.3 Translated instructions

General Instructions for Treatment gains and [Treatment losses]

Welcome! The overall duration of this experiment is approximately one hour. If you have difficulties understanding something now or during the experiment or if you have any questions, please raise your hand and remain seated. We will come to you to answer your question. It is important that you read the instructions and all the explanations on the screen carefully before you start making decisions.

During the experiment, it is forbidden to talk to other participants in the experiment or to communicate with them in any other way. Failing to comply will lead to the exclusion of any payments.

In the following, the general course of the experiment is explained. Today's experiment consists of **three decision-making parts and a subsequent questionnaire**.

In the three decision-making parts, you can earn **Experimental Currency Units (ECU)**. How many ECU you will earn depends on your decisions and decisions made by the other experiment participants. At the end of the experiment, your earnings in ECUs will be converted to Euros. The conversion of your ECUs to Euros will be made in the following way:

1 ECU $= 0.20$ Euros or 100 ECUs$= 20$ Euros.

You will receive the total amount in cash at the end of the experiment **anonymously**. On the next page, you will find further information on the experiment.

[Next Page] You have been allocated to a group of six people to decide between different options that yield **gains [losses]** to be shared among the members of the group. The decision will be made by voting. Therefore, voting decisions will determine how the gains are shared. Your payoffs, and the payoffs of the rest of the voters, are the gains [100 ECUs minus the losses] resulting from the elected alternative.

General procedure:

In each of the three parts you are going to take part in several elections. You are going to decide with five other voters about the outcome of the election. The voting method differs in each decision-making part and will be explained to you in detail on-screen. Each time, there will be 4 alternatives to choose from: A, B, C, and D.

Voting decision:

Your task is to choose between the alternatives in each round according to this election's method. Please notice that you have to make a decision and are not allowed to abstain. Thus, you have to fill a valid ballot in each round.

Payoffs:

At the end of the experiment, **one out of all the rounds will be chosen randomly, and this voting result will be implemented. Thus, your payment will be determined by the winning alternative in the chosen round. It does not matter if you have voted for the winning alternative or not.**

Layout on screen

On the screenshot below [Figure 2 or Figure 3, respectively], you can see how a typical decision-making screen looks like (depending on the voting method, the screen may differ). The numbers on the screenshot are only an example to illustrate some particular elements. The exact numbers on the screen during the experiment will differ from the numbers in the example below. However, the information for the experiment will be displayed as in the example.

- In box "a," on the upper edge of the screen, you see information about the current round. In addition, you are informed about which part of the experiment you are in, and the rules of the voting method that apply to the current round are explained to you again.
- In box "b" you are informed about your type for the current round.
- Table "c" displays the details of the payoffs for each type in the current round. In this example your potential payoffs are the ones of type I.
- In box "d" you can see that the ballot you have to use contains all the alternatives. Depending on the voting method, the ballot can vary a bit. Please fill in the ballot according to the voting method used in the current round. To confirm your decision click on "confirm."

How to read your payoff profile

In this example you would receive the following payment in ECUs:
If alternative A wins, you earn 90 ECUs [100 – 10 = 90 ECUs]
If alternative B wins, you earn 65 ECUs [100 – 35 = 65 ECUs]
If alternative C wins, you earn 60 ECUs [100 – 40 = 60 ECUs]
If alternative D wins, you earn 65 ECUs [100 – 35 = 65 ECUs]

If, for instance, alternative C wins the election, you will earn 60 ECUs [100 – 40 = 60 ECUs]. Your payment does not depend on whether you have voted for C or not. Only the winning alternative matters for your payment in ECUs. Please keep in mind that the possible payoffs in this example will differ from those in the experiment.

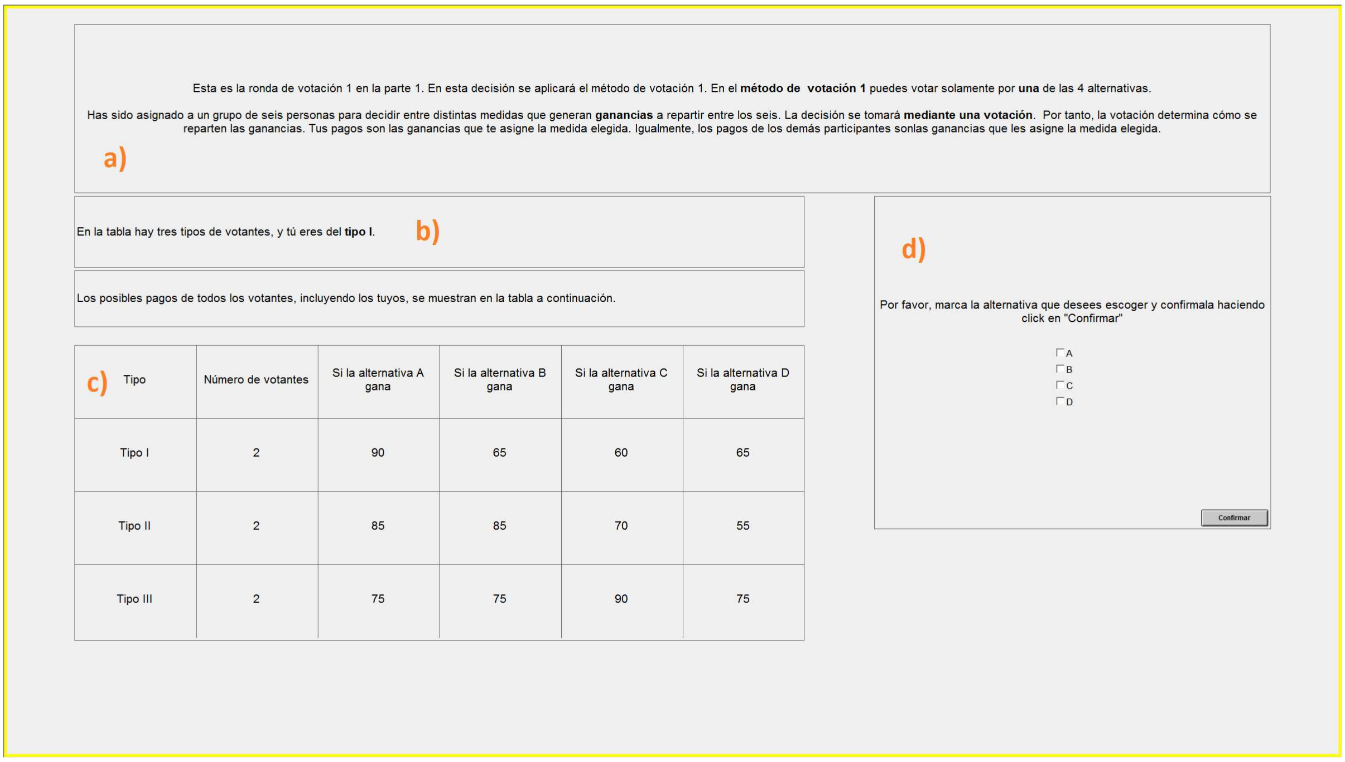

Figure 2. Screenshot for gains of the original Spanish version.

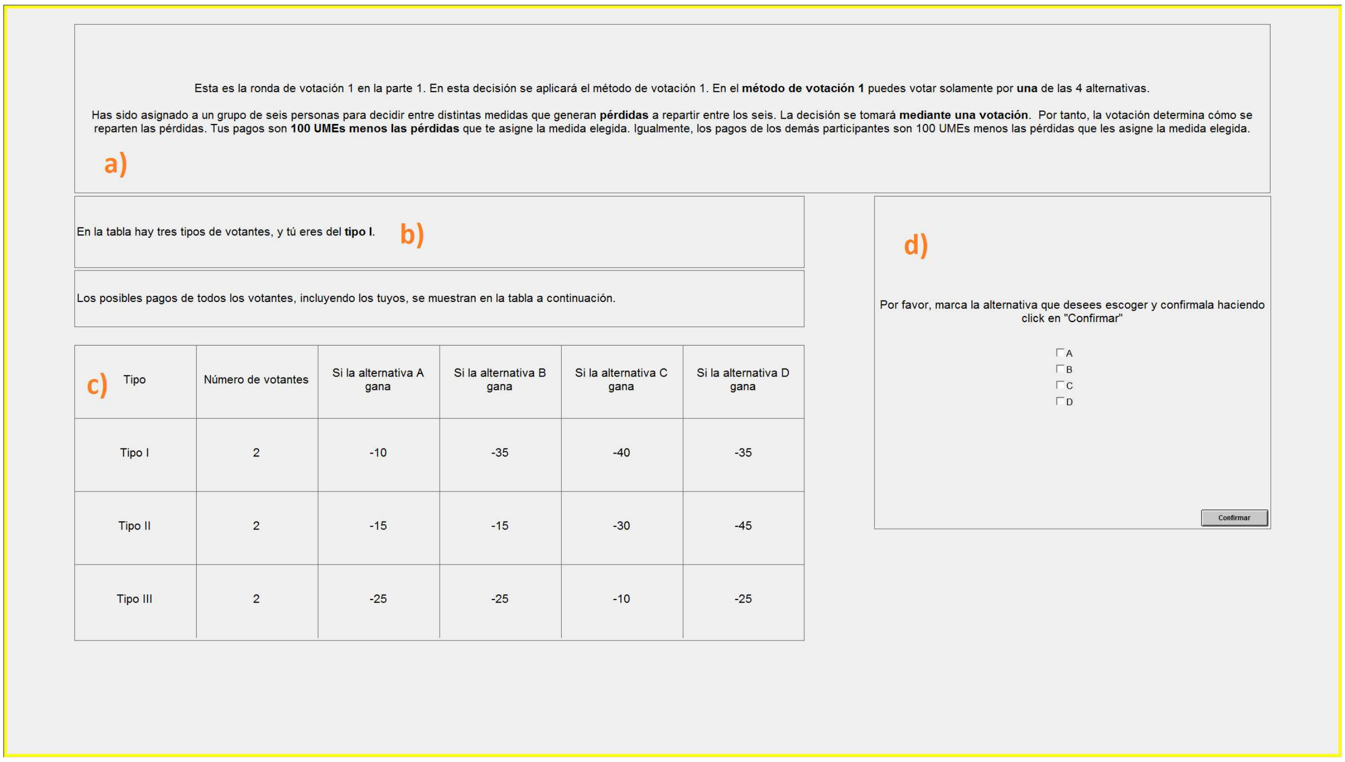

Figure 3. Screenshot for losses of the original Spanish version.

How to read the payoff profile of all six voters
In table c, the payment profiles of all the six voters are displayed. This includes your payoff profile as well. The first column of the table (“type”) tells you the type of the voters, and the potential payoffs for every type are specified in the corresponding row. The second column (“number of voters”) tells you how many voters of every type there are. In this example, you are of type I. Therefore, your potential payoffs are displayed in the first row. In addition, there are other 2 voters whose payoffs are displayed in the second row and 2 voters whose payoffs are displayed in the third row.

In this example, the first row of the table tells you that the 2 voters of type I would get 90 ECUs [100 – 10 = 90 ECUs] if alternative A wins the election, 65 ECUs [100 – 35 = 65 ECUs] if B wins, 60 ECUs [100 – 40 = 60 ECUs] if C wins, and 65 ECUs [100 – 35 = 65 ECUs] in case alternative D wins the election. The second row of the table tells you that the 2 voters of type II would get 85 ECUs [100 – 15 = 85 ECUs] if alternative A wins the election, 85 ECUs [100 – 15 = 85 ECUs] if B wins, 70 ECUs [100 – 30 = 70 ECUs] if C wins, and 55 ECUs [100 – 45 = 55 ECUs] in case alternative D wins the election. The third and last row tells you that the 2 voters of type III would get 75 ECUs [100 – 25 = 75 ECUs] if alternative A wins the election, 75 ECUs [100 – 25 = 75 ECUs] if B wins, 90 ECUs [100 – 10 = 90 ECUs] if C wins, and 75 ECUs [100 – 25 = 75 ECUs] in case alternative D wins the election.

As you can see, there are 6 voters in total. The table displays the possible payoffs of all the voters: **Your own possible payoffs, as well as the payoffs of the other 5 voters** who have to decide jointly with you. Please keep in mind that the payoffs in the experiment will differ from the ones in this example.

Control Questions: Please answer the following comprehension questions. If you have any questions, please raise your hand and remain seated. We will come to you to answer your question.

QUESTION 1: The payment in ECUs I am going to receive in every voting round depends on: (Please circle the correct response)
a) On which alternative wins the election
b) On the alternative I voted for

QUESTION 2: The total payoff I receive for my decisions is computed: (Please circle the correct response)
a) By collecting the payoffs of every decision.
b) At the end of the experiment, it is randomly determined which round is implemented. I will receive my payoffs according to the result of this round.

QUESTION 3: I know the possible payoffs of all the rest of the voters. True or false?
a) True b) False

QUESTION 4: Consider the possible payments of the screenshot displayed in the example on page *[page]*. How many voters have your same possible payoffs? (apart from yourself)
a) Two b) One

QUESTION 5: Consider again the possible payments of the screenshot displayed in the example on page *[page]*. If these are the payoffs of all the voters, and you are of type II, how many ECUs would you get if alternative C wins the election?
a) 60 [100-40 = 60] b) 70 [100-30 = 70] c) 90 [100-10 = 90]

REFERENCES

Alós-Ferrer, C. and Buckenmaier, J. (2021). Alternative Voting Methods Facilitate Compromises in Polarized Societies. Working Paper, University of Zurich

Bolton, G. E. and Ockenfels, A. (2000). ERC: A Theory of Equity, Reciprocity, and Competition. *American Economic Review* 90, 166–193

Brams, S. J. and Fishburn, P. C. (1978). Approval Voting. *The American Political Science Review* 72, 831–847

Charness, G. and Rabin, M. (2002). Understanding Social Preferences with Simple Tests. *Quarterly Journal of Economics* 117, 817–869

Davis, D. D. and Holt, C. A. (1993). *Experimental Economics* (Princeton University Press)

De Sinopoli, F., Dutta, B., and Laslier, J. F. (2006). Approval Voting: Three Examples. *International Journal of Game Theory* 35, 27–38

Fehr, E. and Schmidt, K. M. (1999). A Theory of Fairness, Competition, and Cooperation. *Quarterly Journal of Economics* 114, 817–868

Kagel, J. H. and Roth, A. E. (2016). *The Handbook of Experimental Economics, Volume 2: The Handbook of Experimental Economics* (Princeton university press), 2nd edn.

Laslier, J.-F. (2009). The Leader Rule: A Model of Strategic Approval Voting in a Large Electorate. *Journal of Theoretical Politics* 21, 99–130

Laslier, J. F. (2010). The Basic Approval Voting Game. In *Handbook on Approval Voting*, eds. J. F. Laslier and M. R. Sanver (Springer)

Shayo, M. and Harel, A. (2012). Non-consequentialist Voting. *Journal of Economic Behavior & Organization* 81, 299–313
